# Use of conservative therapy before and after surgery for carpal tunnel syndrome

**DOI:** 10.1186/s12891-021-04378-3

**Published:** 2021-05-26

**Authors:** Juhani Multanen, Mikko M. Uimonen, Jussi P. Repo, Arja Häkkinen, Jari Ylinen

**Affiliations:** 1 Faculty of Sport and Health Sciences, University of Jyväskylä, Jyväskylä, Finland; 2 Department of Physical Medicine and Rehabilitation, Central Finland Central Hospital, Jyväskylä, Finland; 3 Department of Surgery, Central Finland Central Hospital, Jyväskylä, Finland; 4 Department of Orthopedics and Traumatology, Tampere University Hospital, Tampere, Finland

**Keywords:** Carpal tunnel syndrome, Conservative therapy, Rehabilitation, Carpal tunnel release

## Abstract

**Background:**

Conservative therapies are typically offered to individuals who experience mild or intermittent symptoms of carpal tunnel syndrome (CTS) or postoperatively to subjects who have undergone carpal tunnel release. Although long-term studies report mostly positive results for carpal tunnel release, knowledge on the need for conservative treatments following surgery is scarce. The aim of this retrospective cohort study was to examine the use of conservative therapies before and after carpal tunnel releasing surgery.

**Methods:**

Of 528 patients who underwent carpal tunnel release surgery in the study hospital during the study period, 259 provided sufficiently completed questionnaires (response rate 49 %). The patients completed a questionnaire battery including a sociodemographic, medical history and symptom questionnaire, the Boston Carpal Tunnel Syndrome Questionnaire, 6-item CTS symptoms scale and EuroQoL 5D. Frequencies of conservative therapies pre- and postoperatively were calculated. Association between Pain VAS and satisfaction with treatment were examined in patient groups according to the use of conservative therapies.

**Results:**

Of all patients, 41 (16 %) reported receiving only preoperative, 18 (7 %) reported receiving only postoperative, 157 (60 %) reported receiving both pre- and postoperative conservative therapies and 43 (17 %) did not receive any therapies. Preoperative use of conservative therapies was more common in females than males (82 % vs. 64 %; *p* = 0.002), but postoperatively no significant gender difference was observed. The patients who received conservative therapies were younger than non-users in both the preoperative (median age 59 vs. 66; *p* < 0.001) and postoperative (59 vs. 66; *p* = 0.04) phases. The patients reported high satisfaction with their treatment and simultaneous improvement in Pain VAS scores. Those receiving conservative therapies only preoperatively reported the highest satisfaction.

**Conclusions:**

While the use of conservative therapies decreased after surgery, a large proportion of the patients received these adjunct interventions. Patients reported high satisfaction with their treatment one year post surgery. Pain outcome seems to be closely related to satisfaction with treatment.

**Level of Evidence:**

Level III.

## Background

Carpal tunnel syndrome (CTS) is a common complaint causing hand disability in people of working age [1–3]. CTS is suggested to be caused by compression of the median nerve in the carpal tunnel under the transverse carpal ligament due to thickening of the ligament or other structures surrounding the median nerve [4, 5]. In the chronic condition, compression and irritation of the median nerve is followed by tingling, numbness, and pain in the innervation area of the median nerve.

The incidence of CTS has been reported to vary between 105 and 419 per 100 000 person-years peaking around 50 years of age [2, 3, 6, 7]. CTS occurs more commonly in females than males although the difference has been shown to diminish with advancing age [2, 3, 6, 7]. The prevalence of CTS symptoms have been reported to be 7.8 % in the working population [8].

Non-surgical treatment is one option in the management of mild and moderate CTS [9]. Non-surgical conservative therapies, such as splinting, corticosteroid injection, and physiotherapy including manual therapy techniques, such as massage, nerve glide exercises and stretching, have been shown to be effective in treating CTS [9–13]. If symptoms do not respond to non-surgical treatments, CTS is often treated surgically. Surgical treatment of CTS has been shown to be more effective in relieving symptoms than splinting [14]. However, a higher rate of treatment-related complications has been found in surgical than non-surgical treatment [14]. Although symptoms have been reported to decrease significantly post-surgery, knowledge on the need for conservative treatments following surgery is scarce [15]. We found no reports on the prevalence of these therapies after CTS surgery. Studies that have reported on the long-term effectiveness of surgery have not always included possible post-operative therapies [16]. Thus authors who assume they are reporting solely on the effectiveness of surgery may in fact being reporting the results of combined therapies.

The aim of this retrospective cohort study was to examine the use of conservative therapies before and after carpal tunnel releasing surgery.

## Methods

All patients fulfilling the inclusion criteria and who underwent carpal tunnel release surgery (procedure code ACC51) at the Central Finland Central Hospital, Jyväskylä, Finland from January 2016 to February 2017 were invited to participate in the study. Inclusion criteria were age over 18, a moderate to severe primary idiopathic diagnosis of CTS, and sufficient ability to understand Finnish in completing the questionnaires. CTS diagnoses were extracted from the patient records of an extensive physical examination by a surgeon using electroneuromyography (ENMG) or nerve conduction with a handheld measuring device [17]. All patients were required to sign an informed consent according to the Helsinki Declaration before participating in the study. The study protocol was approved by the Ethics Committee of the Central Finland Health Care District (approval number: 15U/2017).

Carpal tunnel release was performed under local anesthesia. An incision was made from Kaplan’s cardinal line to the first wrist crease following the medial palmar crease. After blunt dissection of subcutaneous tissues, the palmar aponeurosis and flexor retinaculum were dissected sharply to reach the transverse carpal ligament. The transverse carpal ligament was then dissected sharply with simultaneous median nerve protection. The landmark of the distal edge of the transverse carpal ligament was fatty tissue parallel to the deep palmar arch where the proximal part of the ligament was released. After complete release of the transverse carpal ligament, the wound was closed using nonabsorbable sutures.

On discharge, all patients received printed instructions. These had earlier been reviewed with the patient by a nurse and physiotherapist and included information on wound healing, medications, and rehabilitation exercises. Patients were instructed on a postoperative rehabilitation exercise program to be performed daily at gradually increasing intensity until use of the operated hand returned to normal. Patients were instructed to move their fingers and wrist immediately after the operation. Extreme movements of the wrist were to be avoided until removal of sutures from the wound. The operated hand was limited to carrying a maximum weight of 1.5 kg for the first 4 weeks after the operation. The movement exercises were instructed to begin on the first postoperative day and included finger stretching and the moving of all fingers and wrist. All exercises were performed four times a day for 4–5 weeks or until the hand was symptomless. Length of sick leave was 4–5 weeks depending on the patient’s occupation. Supervised physiotherapy was not prescribed as a routine. If the patients encountered any problems during rehabilitation, they were advised to contact their physiotherapist in primary health care. Acetaminophen or a non-steroidal anti-inflammatory drug was prescribed against postoperative pain.

Invitation letters along with questionnaires and a blank informed consent were mailed to patients one year after surgery. The questionnaire battery included a sociodemographic and a medical history questionnaire with items on mean hand and wrist pain intensity during the last week before surgery and after surgery on a visual analogue scale (VAS; 0–100 mm). Time with symptoms before surgery and therapies received before and after surgery were elicited with direct questions and multiple choice questions with a single or multiple response options. Satisfaction with treatment was investigated with a question “Did the surgery improve the condition of your hand or wrist?” with a five-point response scale from “No improvement” to “Eliminated symptoms completely” (scores 0–4). In addition, the Boston Carpal Tunnel Syndrome Questionnaire (BCTQ) [18–20], 6-item CTS symptoms scale (CTS-6) [21] and EuroQoL 5-dimension questionnaire (EQ-5D-5 L) [22] were included in the questionnaire battery. The EQ-5D-5 L index ranges from − 0.011 to 1 in a Finnish reference group, with a higher score indicating higher health-related quality of life.

### Statistical analyses

The results are presented as frequencies, means with standard deviations (SD) or medians with interquartile ranges (IQR). Change in the frequency of conservative therapies pre- and postoperatively and change in Pain VAS scores were calculated. The association of the use of conservative therapies pre- and postoperatively with age was examined using the Mann-Whitney U-test, and the association with gender was examined using a chi-square test. Pre- and postoperative Pain VAS scores were compared using the Mann-Whitney U-test. Patients were further divided into five subgroups according to the response categories of the subjective improvement question, and the change in Pain VAS scores was examined in each of the five subgroups. Differences in Pain VAS scores and satisfaction with the use of conservative therapies were examined in the patient subgroups using the Kruskal-Wallis test. R version 3.6.1 statistical software was used in statistical analysis (R, 2019).

## Results

A total of 528 patients underwent carpal tunnel release surgery during the study period. Of these, 259 returned sufficiently completed questionnaires, yielding a response rate of 49 % (Table 1). Females formed the majority (66 %) in the sample and mean patient age was 62 (range 26–96). Approximately half of the patients were working at least part-time while the other half were retired. Symptomatic time before surgery varied widely (IQR 4–36 months).

**Table 1:** Sociodemographic and clinical characteristics of the patients

	*N* = 259
Days between surgery and administration of questionnaire battery, median (IQR)	408 (371–490)
Female, n (%)	172 (66)
Age, years, mean (SD)	62 (15)
BMI, mean (SD)	29 (5)
Smoking, n (%)	30 (12)
Days off working due to CTS, median (IQR)	
Before surgery	0 (0–28)
After surgery	28 (21–42)
Occupation, n (%)	
Working	114 (44)
Retired	125 (48)
Other	20 (8)
Time with symptoms before surgery in months, median (IQR)	
Pain	18 (9–36)
Numbness	12 (6–36)
Muscle weakness	12 (4–24)
BCTQ scores 1 year after surgery, median (IQR)	
Symptoms severity scale	1.5 (1.2–2.3)
Functional status scale	1.4 (1.0–2.0)
CTS-6 score 1 year after surgery, median (IQR)	1.3 (1.0–2.2)
EQ-5D-5 L index 1 year after surgery, median (IQR)	0.67 (0.52–0.73)

*IQR* interquartile range; *SD* standard deviation; *CTS* carpal tunnel syndrome; *BCTQ* Boston Carpal Tunnel Questionnaire; *CTS-6* Carpal Tunnel Syndrome 6-item questionnaire; *EQ-5D* EuroQoL 5-dimension questionnaire

### Use of conservative therapy

Overall, 41 (16 %) patients reported receiving only preoperative, 18 (7 %) reported receiving only postoperative, 157 (60 %) reported receiving both pre- and postoperative conservative therapies and 43 (17 %) did not receive any therapies. The most commonly received preoperative therapies were splinting, stretching, pain medication and massage. The most commonly used postoperative treatments were stretching and pain medication (Table 2). Use of splints was minimal post-surgery.

**Table 2:** Conservative treatments for carpal tunnel syndrome pre- and postoperatively

	Number of patients, n (%)			
	No	Only preoperatively	Only postoperatively	Pre- and postoperatively
Corticosteroid injection	238 (92)	19 (7)	1 (0)	1 (0)
Volar splint	161 (62)	83 (32)	2 (1)	13 (5)
Dorsal splint	223 (86)	28 (11)	2 (1)	6 (2)
Stretching	93 (36)	33 (13)	33 (13)	100 (39)
Acupuncture	248 (96)	10 (4)	0 (0)	1 (0)
Pain medication	109 (42)	37 (14)	28 (11)	85 (33)
Wrist massage	170 (66)	40 (15)	19 (7)	29 (11)
Neck massage	216 (83)	23 (9)	9 (3)	11 (4)
Any conservative treatment	43 (17)	41 (16)	18 (7)	157 (61)

**Fig. 1:**
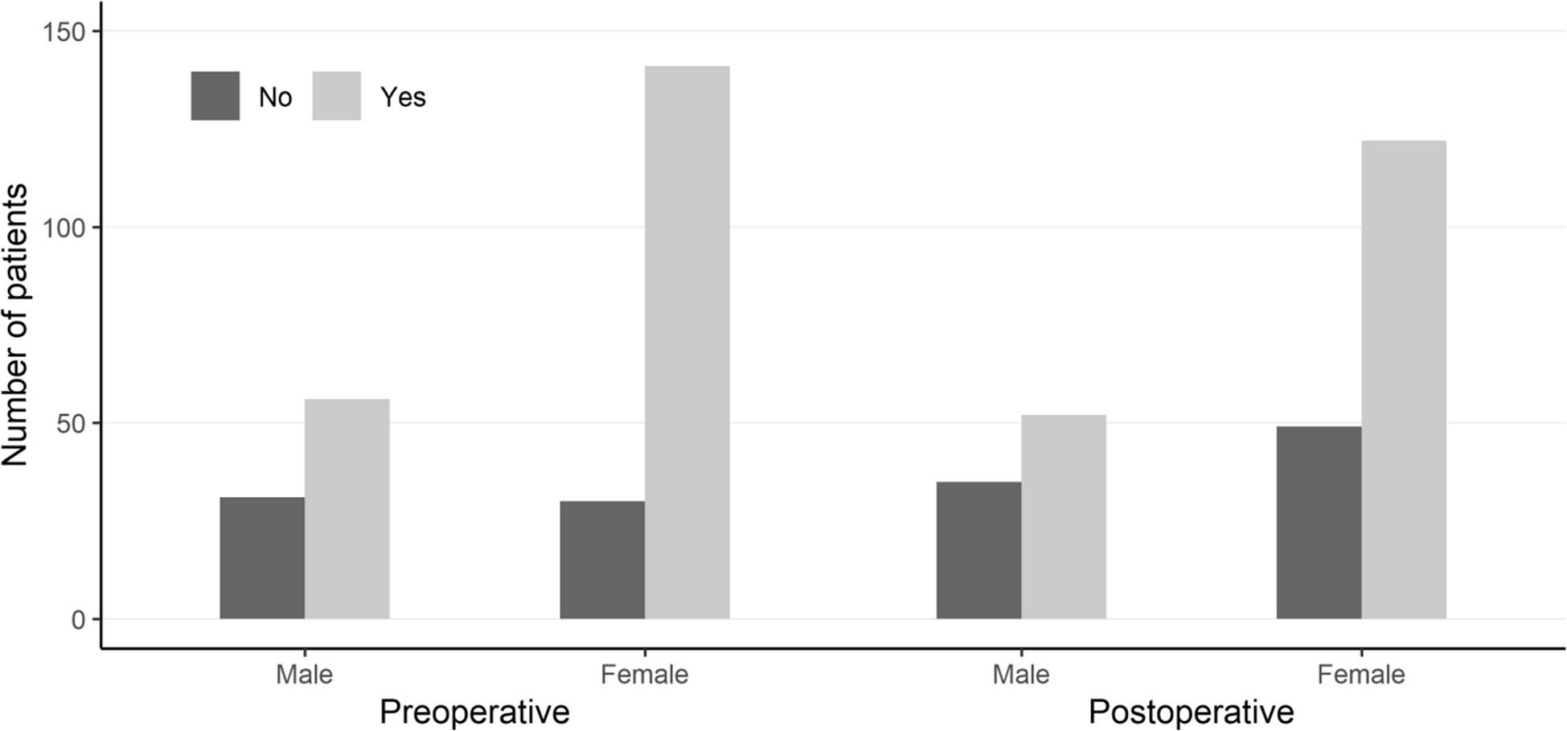
Use of conservative therapies in males and females

A statistically significant gender difference was found in the use of preoperative conservative therapies (82 % of females vs. 64 % of males; p = 0.002) whereas postoperatively the difference did not reach statistical significance (71 % of females vs. 60 % of males; *p* = 0.08; Fig. 1). The patients who received conservative therapies were younger than non-users both preoperatively (median years of age 59 vs. 66, respectively; *p* < 0.001) and postoperatively (59 vs. 66; *p* = 0.04).

### Satisfaction with treatment

**Fig. 2:**
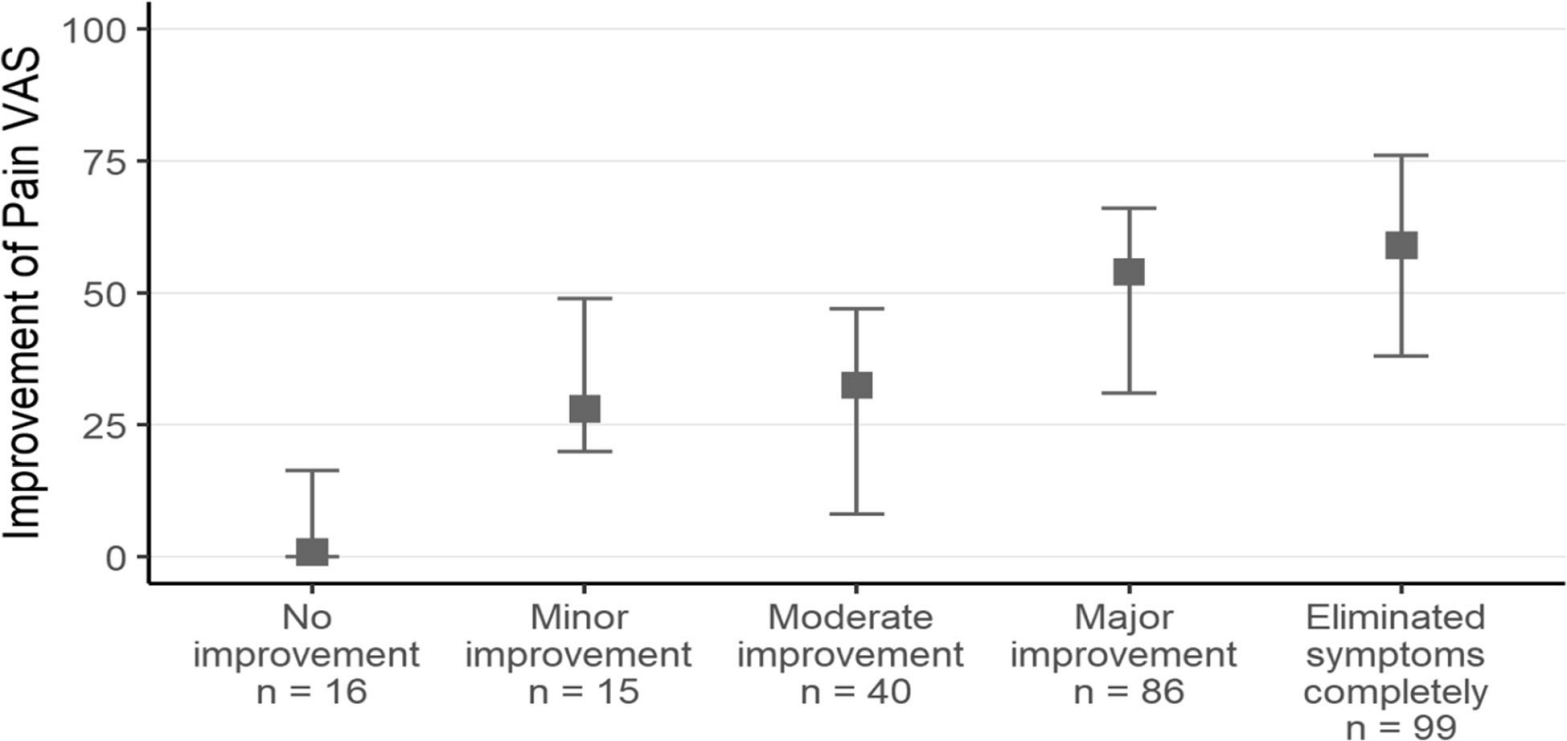
Distribution of reported improvement in pain on visual analogue scale (VAS; 0-100 mm) at one-year follow-up after carpal tunnel surgery. The square denotes median and whiskers denote interquartile range

Pain VAS scores improved significantly from the pre- to one-year postoperative measurements (median 63.5 vs. 7.5, *p* < 0.001). In 38.1 % of patients, symptoms were completely eliminated after surgery, and an additional 33.1 % reported major improvement in the condition of their hand (Fig. 2). Only 6.2 % of patients reported no improvement after surgery. In the patients who reported that their symptoms were completely eliminated, the Pain score had diminished by 58 (IQR 33–74) points and in the patients who reported a major improvement the Pain VAS score had diminished by 51 (IQR 31–66) points. In the “No improvement” group, the Pain VAS scores remained the same (median change 0, IQR 0–10). The Spearman correlation coefficient between the reported improvement in hand condition and the improvement in Pain VAS was 0.35 (*p* < 0.001). In females, both satisfaction with treatment and improvement in Pain VAS were higher than in males. The patients who were working were more satisfied than retirees (*p* = 0.017) and also reported higher Pain VAS improvement than retirees (*p* = 0.014).

The patients who received conservative therapies preoperatively only (median satisfaction 3) reported higher satisfaction with treatment than those in the other patient groups (2.3 in only postoperatively; 2 in both pre- and postoperatively; and 2 in non-users) (*p* = 0.03).

## Discussion

In the current cohort, the use of different conservative therapies decreased considerably after carpal tunnel release surgery for primary CTS. Nevertheless, 67 % of the patients reported that they had undergone interventions within a year after surgery. As postoperative rehabilitation was not a routine protocol, the finding may represent variation in the management decisions of treating physicians and/or a genuine need of patients for further interventions. According to a systematic review of the few studies available on postoperative rehabilitation, evidence on the benefits of these interventions is controversial [15]. Thus, while they were not common practice in postoperative rehabilitation, symptomatic patients sought help from these therapies.

Non-surgical approaches have been recommended as a first-line treatment in patients with mild and moderate symptoms [14, 23]. The present findings show that while the majority of patients underwent conservative therapy before surgery, this did not include all of those with mild or moderate symptoms. Reported preoperative non-surgical treatments do not give the full picture. Patients who do not benefit from nonsurgical treatment are recommended for surgery. However, as the prevalence of subclinical CTS symptoms is relatively high in the general population, with only one-fifth meeting the diagnostic criteria of CTS, the present findings favoring surgery might not apply to all patients with CTS symptoms [14]. In addition, patients who experienced sufficient symptom reduction from non-surgical treatment were not included in the present sample.

The study showed that a considerable need for treatments remained after surgery. Although a large proportion of the patients underwent conservative therapies after surgery, they generally reported high satisfaction with their surgical treatment. Overall, 71.2 % of the patients reported a major improvement or complete elimination of symptoms in the affected hand. In addition, perceived pain decreased considerably after surgery. Satisfaction with surgery seemed to be concomitant with the decrease in pain. In addition, a significant, though low, correlation was found between the improvement in the Pain VAS score and reported satisfaction with surgery. These findings are in line with previous studies reporting good outcomes after CTS surgery [14].

In the present sample, the majority of the patients were female, and mean patient age was 61.9 years, as similarly found in previous epidemiological studies [2, 3, 6]. CTS has been reported to occur more frequently in females, although the difference has been found to diminish with age [2, 3, 6]. In our study, females and employed patients reported better outcomes than males or retirees one year after CTS surgery. This might be explained by the finding that the females had received more conservative therapies than males and that the patients who received conservative therapies were younger than non-users. However, this study did not include patients who had recovered and did not need surgery after conservative therapy. Among younger patients, a more active treatment approach enabling them to regain their ability to work may be warranted. The need for postoperative conservative therapies may be due to more severe complaints which surgery alone does not sufficiently remedy [24]. The demand for postoperative conservative therapies may also reflect unsuccessful or complicated surgery [25]. Further studies are needed to illuminate the causality behind these findings.

To the best of the authors’ knowledge, this is the first study to report on the incidence of postoperative conservative therapies and the change in the incidence of such interventions between pre- and postoperative phases. The main limitation of this study is the retrospective design and relatively low response rate (49 %). The patients completed the questionnaires one year after surgery, and thus information on the preoperative state was collected postoperatively. This might have predisposed the answers to recall bias. However, the questions on preoperative interventions were designed to mitigate recall bias by offering direct and dichotomous response options (“yes” or “no”). With regard to Pain VAS, direct comparison of the pre- and postoperative phases offered opportunity to observe perceived change. The reasons why about half of the invited patients did not respond to the questionnaires remain unknown. It can be assumed that nonrespondents would experience less discomfort and symptoms at the one-year follow-up after surgery whereas morbidity would be higher in respondents, thereby explaining their willingness to participate. Nevertheless, the response rate of 49 % in our study is comparable to the 42 % reported by Schwartzenberger et al. (2020) for a mail survey of the BCTQ at 1 year after CTS surgery [26].

## Conclusions

The present study shows that use of conservative therapies decreased after carpal tunnel release. Although the patients reported high satisfaction with the surgery, a large proportion of the patients were continuing with conservative therapies one year after surgery. It is important to inform patients of this, as it may affect their acceptability of conservative therapies in an early stage.

## Data Availability

The datasets generated and analyzed during the current study are available from the corresponding author on reasonable request.

## Abbreviations

ACC51: Code for decompression and freeing of adhesion of median nerve
BCTQ: Boston Carpal Tunnel Questionnaire
CTS: Carpal tunnel syndrome
CTS-6: Six-item carpal tunnel symptoms scale
ENMG: Electroneuromyography
EQ-5D-5L: EuroQol five-dimensional questionnaire with five-level version
IQR: Interquartile range
SD: Standard deviation
VAS: Visual analogue scale
